# Improving TB case notification in northern Uganda: evidence of a quality improvement-guided active case finding intervention

**DOI:** 10.1186/s12913-018-3786-2

**Published:** 2018-12-12

**Authors:** Esther Karamagi, Simon Sensalire, Martin Muhire, Herbert Kisamba, John Byabagambi, Mirwais Rahimzai, Frank Mugabe, Upenytho George, Jacqueline Calnan, Dejene Seyoum, Estella Birabwa

**Affiliations:** 1 University Research Co., LLC, USAID Applying Science to Strengthen and Improve Systems (ASSIST) Project, USAID, Suite 611, Level 6, BMK House, Plot 45, Nyabong Road, P.O. Box 28745, Kampala, Uganda; 2 Ministry of Health, Kampala, Uganda; 3 United States Agency for International Development (USAID), Kampala, Uganda

**Keywords:** Tuberculosis, High risk population, Screening, Active case finding

## Abstract

**Background:**

Strategies to identify and treat undiagnosed prevalent cases that have not sought diagnostic services on their own, are necessary to treat TB in patients earlier and interrupt transmission. Late presentation for medical services of symptomatic patients require special efforts to detect early and notify TB in high risk populations. An intervention that combined quality improvement with facility-led active case finding (QI-ACF) was implemented in 10 districts of Northern Uganda with the highest TB burden to improve case notification among populations at highest risk of TB.

**Methods:**

Using QI-ACF intervention approach in 48 facilities, we; 1) targeted key vulnerable populations, 2) engaged district and facility teams in TB systems strengthening, 3) conducted systematic screening and diagnosis in vulnerable groups (people living with HIV, fishing communities, and prisoners), and 4) trained health workers on national x-ray diagnosis guidelines for smear-negative patients. Facility-led QI-ACF meant that health care providers identified the target population, mobilized and massively screened suspects, and addressed gaps in documentation. Chest X-ray diagnosis was promoted for smear-negative TB among those suspects whose sputum examination was negative. The effect of the intervention on case notification was then assessed separately over the post intervention period.

**Results:**

Over all TB case notification in the intervention districts increased from 171 to 223 per 100,000 population between the baseline months of October–December 2016 and end line month of April–June 2017. TB patient contacts had the majority of TB positive cases identified during active case finding (40, 6.1%). Fishing communities had the highest TB positivity rate at 6.8%. Prisoners accounted for the lowest number of TB positive cases at 34 (2.3%).

**Conclusion:**

Targeting should be applied at all levels of TB intervention to improve yield: targeting districts and facilities with the lowest rates of case notification and targeting index patient contacts, HIV clients, and fishing communities. Screening tools are useful to guide health workers to identify presumptive cases. Efforts to improve availability of x-ray for TB diagnosis contributed to almost half of the new cases identified. Having all HIV patients who were eligible for viral load provide sputum for TB screening proved easy to implement.

**Electronic supplementary material:**

The online version of this article (10.1186/s12913-018-3786-2) contains supplementary material, which is available to authorized users.

## Background

Sustainable Development Goal (SDG) 3 aims at ending deaths from and transmission of tuberculosis (TB) by 2030. In 2016, 1.4 million people died from TB, with 95% of these deaths in low- and middle-income countries like Uganda [1]. Unfortunately, the rate of decline of TB incidence is so slow that if the current situation remains unchanged, it will take up to 2182 to reach the WHO End TB targets [2]. Despite the global magnitude of the TB problem, case notification rates have stagnated since the late 2000s and 3 million incidence TB cases are estimated to remain undiagnosed or not notified each year, thus contributing to a significant shortfall in the actions and investments needed to end the global TB epidemic [3]. Worldwide, the case fatality rate for TB is estimated at 55% for untreated persons and 15% for persons receiving treatment but varies widely by region and level of socioeconomic development [3].

In Uganda, it is estimated that annually, 136 new smear-positive cases of TB occur per 100,000 population; and the incidence of TB in all forms in Uganda is estimated at 330 cases/100,000 population [4]. Despite the disease burden, the number of notified cases in the country is still low at 235/100,000 compared to the expected number of 253/100,000 cases [4]. Despite achievements of the National TB and Leprosy Control Program (NTLP) under the Health Sector Strategic Plan (HSSP) II, such as adopting the ‘Global plan to STOP TB strategy’ (2006–2015), the problem of low case notification persists [4]. Post-conflict Northern Uganda is not exceptional. According to literature, armed conflicts affect disease control programs by destroying infrastructure and health systems as well as interrupting patient’s access to health care [5]. It is estimated that the 10 intervention districts of Northern Uganda have a TB case notification of 171/100,000, much lower than its expected TB prevalence [4].

The changing epidemiology of TB in Uganda is characterized by a disproportionate burden of disease in certain vulnerable populations such as prisoners, HIV-seropositive persons, fishing communities, contacts of TB patients, migrants/refugees, alcoholics, and socially and economically disadvantaged groups, especially in congregated urban settings [6]. Efforts towards preventing and curbing the spread of TB in these vulnerable populations are affected by the delays in diagnosing suspects and poor adherence to anti TB drugs [6]. TB services do not target groups of people at a high risk of TB and drug resistance [7]. Several studies have found that TB burden varies in different population categories due to different risk factors. This implies that, certain categories of populations most at high risk should be targeted with specific interventions that are also customized to their settings [8].

Apart from the increased risk of exposure to *Mycobacterium tuberculosis* (MTB), high risk groups would progress to active disease once they are infected as a result of a weakened immune system more so when TB is diagnosed after a long time. The undiagnosed cases among the high risk groups will therefore, perpetuate ongoing TB transmission in their communities [8]. People infected with HIV are often infected with tuberculosis, as their immune systems are severely compromised [9]. In prisons, TB is a major public health concern due to the presence of enabling factors for transmission such as overcrowding and inadequate medical services [10]. TB contacts in the household and other close contacts of persons with active TB (TB contacts) have a high risk of becoming infected [1].

Quality improvement (QI) is an approach which identifies and addresses failures in the health system that result in poor patient outcomes by actively engaging health care workers in analyzing gaps in care processes, testing changes to close the gaps, and monitoring the results from implementing the tested changes [11]. In diverse settings, including Uganda, QI has been shown to improve health outcomes, including GeneXpert utilization for detection of TB [11, 12]. Given the different contexts and approaches for TB response, successful interventions need to be documented and compared to inform policies and practice. Various strategies to improve TB case-finding, such as improving health communication, engaging all care providers, improving diagnostic sensitivities, and strengthening the health system are being implemented. While these activities are important, QI-guided active case-finding remain unexplored and yet could have the potential of increasing TB case finding [13].

This paper describes a QI-based intervention with a research component used to document and explain changes in TB case notification in 10 districts in Northern Uganda among high-risk populations. The intervention engaged district leaders and facility-level health workers in targeting, systematic screening and diagnosis within vulnerable groups and taking steps to improve adherence to guidelines for smear-negative patients. Targeting, a commonly used technique in public health, uses a specialized health intervention approach for a specific group of people rather than the general population [14]. This helps to identify TB risk groups and prioritize them [15]. The intervention hypothesis was that facility-led QI-ACF would help identify who is most at risk for TB and therefore, lead providers to perform systematic screening and diagnosis within vulnerable groups. Contact investigation is also necessary for identifying persons at risk of contracting TB. It’s one of the strategies for increasing case detection and involves examining and treating persons who are close contacts with patients having infectious TB [16]. Active case-finding among contacts of patients with TB rather than the general population helps to find contacts that will be infected [16].

In many sub-Saharan countries with where a large number of suspects needs to be evaluated to detect a TB patient, diagnostic procedures are not strictly followed leading to under-diagnosis [17]. TB suspects with MTB not detected and smear-negative patients should undergo further clinical evaluation and/X-ray in accordance with the NTLP algorithm for diagnosis and management of TB algorithm for presumptive TB cases [4]. Like any other public interventions, systems strengthening related with quality and timely data is important for decision making in TB interventions [14]. Although routine TB services are necessary for case management, they are not accessible by the poor and high risk groups in communities [9]. QI-guided active case-finding (QI-ACF) is being implemented as an approach for improving case detection in situations where services are not fully accessible. Yet, there is limited information on the effect of QI-ACF on case notification [2].

### Description of the intervention of quality improvement-active case-finding model for TB

**Fig. 1:**
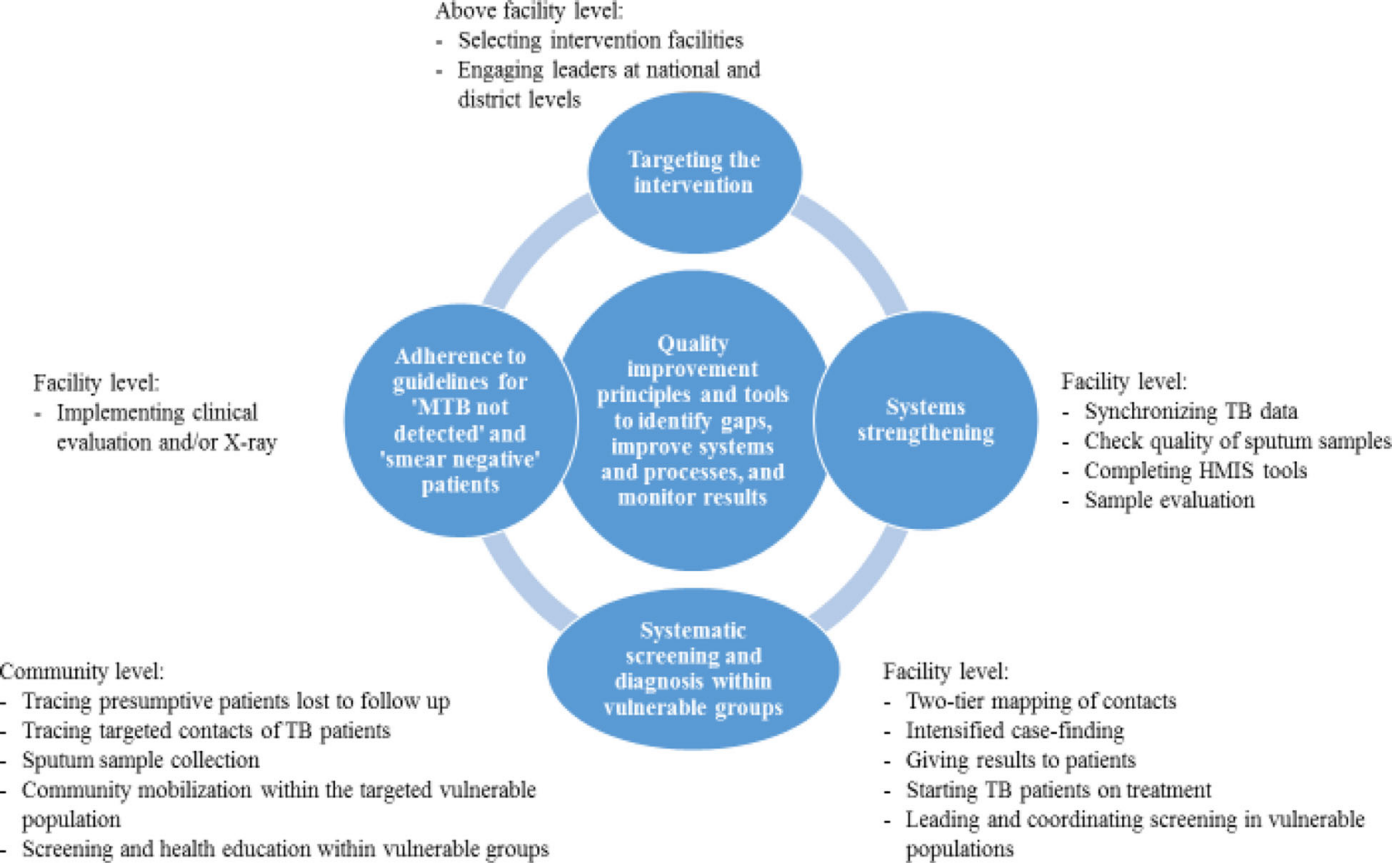
QI-ACF intervention components

Facility-led QI-ACF was led by facility-based improvement teams and was grounded in QI tools and methods, such as Pareto charts, process mapping, cause-and-effect analysis, and the plan-do-study-act cycle. As depicted in Fig. 1, it encompassed four main strategies: 1) targeting, 2) systems strengthening, 3) systematic screening and diagnosis within vulnerable groups, and 4) improved adherence to guidelines for ‘MTB not detected’ and ‘smear-negative patients’. These four components, implemented with the use of quality improvement science, represent the theory of change behind the intervention. The intervention involved key stakeholders and coordinated activities at three levels: above facility, facility level, and community level. An experienced improvement coach worked with facilities to form QI teams to identify and quantify gaps in systems and processes**,** test small changes and new ideas at the facility level to improve case detection and notification, and use data to monitor improvement.

Facility-level QI plans addressed the following processes: identification and screening of targeted vulnerable populations, evaluation of presumptive TB cases, HIV testing of newly diagnosed TB patients, documentation of newly diagnosed TB patients in the TB register, contact tracing of TB patients, X-ray verification of smear-negative presumptive cases, addressing drug stock-out, supply challenges, and quality of sputum samples. Illustrative indicators that facilities used to monitor improvement included; proportion of TB patients whose contacts are screened for TB, proportion of patients eligible for X-ray who had an X-ray done, proportion of presumptive TB cases evaluated for TB, proportion of new TB-positive patients enrolled in the TB register, proportion of people in targeted vulnerable populations screened for TB, proportion of new TB-positive cases tested for HIV.

We designed a program around these parameters and borrowed from other documented intervention models such as the onion model [18]. We describe the notification trends following the intervention by estimating case notification before, during, and after the intervention period. We also assessed TB positivity rates among the high-risk population groups and collected health worker perceptions following the QI-ACF intervention. TB positivity rate was operationally defined as the proportion of TB presumptive (or TB suspects) whose samples were evaluated, who were confirmed positive for TB. The research component of the intervention documented and examined the effects of a facility-led QI-ACF intervention to systematically improve TB notification in Northern Uganda.

### Targeting the intervention

Over 3643 TB cases were missing in the 15 districts of Northern Uganda. We selected the ten (10) intervention districts using the following criteria: 1) contributing to 80% of the missing TB cases, as estimated using TB case notification rate of 253/100,000 and district population from the District Health Information System (DHIS2); 2) presence of vulnerable population of prisoners and fishing villages; and 3) availability of functional X-ray for diagnosis of TB. The districts selected for intervention, based on the three criteria, were: Apac, Oyam, Alebtong, Kole, Amuru, Agago, Dokolo, Amolatar, Kitgum, and Gulu. Together, these districts had a total of 2812 TB patients missing, accounting for 77% of the estimated missing TB cases in Northern Uganda. Given the mechanisms of TB transmission and available data, four key vulnerable populations were targeted: 1) Contacts of TB index patients, 2) HIV-infected patients, 3) prisoners, and 4) fisher folks. A total of 48 intervention health facilities were selected on the basis on one or more criteria. Thirteen sites were selected for contact tracing of index clients; 21 were selected because they served large numbers of HIV-positive clients; 4 were selected because they served fishing communities; and 10 were selected because they had a functional X-ray machine or within 5 to 7 km radius from the functioning X-ray points. The five non-intervention districts that were Amolator, Kitgum, Otuke, Lamwo, and Nwoya with baseline case notification of 125/100000.

### Systems strengthening

#### Engaging district leadership

The NTLP Program Manager and team provided strategic leadership for this work through consultative meetings and direct involvement, leading activities. A focal person for this intervention was assigned within the NTLP and, together with the NTLP Program Manager, officiated at national and sub-national meetings to roll out the intervention, ensuring that changes being tested were in line with national and international guidelines. Although ACF activities began in December, pre-intervention activities involving planning meetings and trainings occurred earlier in November 2016. Training focused on x-ray and clinical diagnosis for TB with participants chosen on the basis of being involved in TB management and diagnosis, including; DHOs, district TB and Leprosy Supervisors, laboratory focal persons, representatives from health facilities and District-level Secretaries for Health from the 10 intervention districts. The participants were carefully selected by virtue of their roles and potential to strengthen systems and processes for TB case detection, diagnosis, and case management. The meeting was co-facilitated by the MOH NTLP and the USAID-funded technical assistance project supporting system strengthening in Northern Uganda, the USAID ASSIST Project. It involved a training in various aspects of TB, explaining the components of the intervention, and drafting district-specific and integrated work plans that would contribute to continuity of TB work.

Earlier to the stakeholder meeting, further engagement with district leaders was done through formal meetings during district visits in June 2017. Every visit to a district began with a courtesy call to district health officer’s (DHO) office to provide support to the team and join them in active TB case-finding wherever they have planned to conduct it. On many occasions, the district office would avail staff scheduled to conduct active case-finding. The review meeting in June 2017 was held to share progress regarding TB active case-finding and develop plans on how to take to scale best practices realized in the intervention facilities within the district and introduce them in other facilities that were not directly involved in TB active case-finding.

#### Improving quality of TB data

To ensure all TB cases detected were notified, facility teams were guided to improve the accuracy of TB records by synchronizing data between the TB unit and the laboratory registers using a specific tool. The improvement changes applied here were adapted from a QI collaborative that improved the quality of data for malaria in another part of Uganda [19]. Synchronizing records helped to: 1) prevent poor quality data, 2) promote re-checking of the data, and 3) encourage correction of discrepancies. Synchronizing was done by comparing data on newly diagnosed TB cases from the national DHSI2 for each health facility with numbers recorded in the facility’s registers. Health facility teams usually checked the laboratory register, counted the number of TB cases that tested positive the previous quarter or month, and noting the patient’s name, age, address, and date of each test done. They also used the TB unit register and recorded the same TB cases identified in the laboratory register and compared them to patients listed. Tallies of the cases were made for those found in the laboratory register but were not in the TB unit register, indicating unnotified cases. Such cases were then recorded in the unit TB register and then actively followed up so they were started on treatment. In addition, follow-up was done by asking the Village Health Teams (VHTs) to trace the case in the community or make a phone call in case the telephone contact was recorded in the registers. If the case was an HIV co-infected person, tracing was done upon the patient’s return for HIV care and the patient started on TB treatment. When there were discrepancies between the number of TB cases registered in the TB unit register the previous quarter and those reported into the DHSI2, the DTLS was contacted to assign such cases so they are reported the next quarter.

### Systematic screening and diagnosis within vulnerable groups

#### Contacts of TB index patients

Two-tier mapping was done to trace contacts of TB patients. First, health facility QI teams used the TB unit register to select villages that have the highest number of TB patients registered in care. These were prioritized for contact tracing. Second, at some facilities, the TB focal person developed a map of the facility catchment area clearly indicating homes of TB patients to allow for easy tracking of TB patients’ contacts. On the map, patients’ treatment numbers were indicated and not names to protect confidentiality. After mapping out patient locations, contact tracing was conducted involving a team of a laboratory technician, the TB focal person, a nurse and a VHT who helped in mobilizing and gathering individuals in the community for screening and identifying suspects. VHTs were selected on the criteria that; they are community resourceful person, respectable in the community, able to write and read and have been involved in health care provision. The health workers were tasked with: i) filling out the ICF tool, ii) giving instructions on sputum sample collection, iii) receiving the sample after the suspect has produced it, confirming the right quality and quantity, and packaging it, and iv) filling in the GeneXpert request form.

Samples collected from the community were delivered to laboratory hubs for evaluation. In circumstances where the samples were many, they were distributed to other GeneXpert testing sites so that results could be ready in a short period of time. Once ready, results would be communicated back to the respective facilities either by phone by calling the site where they originated and reading details of the positive TB case or taking a picture and sending to the TB unit that sent the samples for testing. Positive patients were given appointments consistent with the expected date of results or contacted once results were received by the facility using their telephone numbers taken at time of collecting samples or through VHTs and enrolled on treatment. Contact tracing was mostly done over the weekend since contacts were more likely to be found at home then. Prior to the intervention, screening and diagnosis was done using a national TB algorithm. This algorithm starts with presumptive TB cases. We therefore designed algorithms for screening close contacts of TB patients (Additional file 1), HIV-positives, and prisoners to guide the processes before the presumptive TB cases are identified.

#### TB screening in prisons

As part of active case-finding, prisons were targeted for TB screening in both Acholi and Lango sub regions. The district and health facility staff took the lead in organizing screening visits to the prisons. Permission was sought from the prison leadership and agreed on the date and time when prisoners would be screened for TB. At the prison, the team worked with one prison officer to mobilize / organize prisoners during sample collection. As described earlier under screening for contacts of TB index cases, the same number of staff participated in sample collection after which the samples were sent to the GeneXpert sites for testing and results returned as soon as it was possible. Both central and farm prisons were targeted in Northern Uganda. TB prevention and health education was part of the screening exercise, providing prisoners and the prison management with information on spread of TB and signs and symptoms (S&S). We developed an algorithm for TB screening in congregate settings (i.e., including people in prisons, slums, among others), shown in the Additional file 2.

#### TB screening among people living with HIV

Prior to the intervention, TB symptom screening among HIV clients was not routinely performed in the intervention districts, a practice which would have contributed to missing TB cases among the HIV clients. As part of the intervention, we developed and piloted an algorithm (Fig. 4) for systematic TB screening in the HIV-positive population. The algorithm (Additional file 3) emphasized symptom screening at every visit to the HIV clinic and sputum GeneXpert testing at the time of viral load (VL) testing for all clients eligible for VL testing, irrespective of whether they were asymptomatic, to increase opportunities for utilizing the GeneXpert technique of TB diagnosis since the TB survey findings had shown TB S&S in only 50% of TB cases. Initially, six sites were purposively selected for piloting the algorithm based on the readiness of staff to test the use of the algorithm; later it was used in all 48 intervention sites. The tool was used in the HIV clinics for all HIV-positive clients to aid TB screening. Those due for VL testing were guided to produce sputum, which was sent for GeneXpert testing while the rest of the HIV patients were screened using the intensified case-finding tool. Tagging GeneXpert to viral load testing was intended to increase HIV-positive patients’ opportunity to get GeneXpert testing while minimizing overloading the care system. This provided for integration of TB screening among the HIV clients and took advantage of the time clients presented at the health facility so they would not have to return on a different clinic day.

#### TB screening among the fishing community

The congested living conditions among the fishing communities guided the promotion of systematic screening in this sub-population. This was further supported by information obtained from interactions with health care providers. At a health facility in Apac District for example, health workers commented that the majority of TB clients usually came from the landing sites. The project team then developed a plan to screen at landing sites, including scheduling heath workers to participate in the activity and liaising with the VHTs at each of the landing sites so they could mobilize the community for TB screening. The VHTs also planned with the fishermen so screening was done at a time when they were not on the water fishing.

The procedure to screen for TB at the landing sites included: Engaging the health workers (facility in charge, TB focal person, laboratory technician, and a health assistant) from the health facility serving the landing site of interest to identify community resource persons to work with. Such community resource persons included the sub-county health worker, VHTs from the fishing community, and the Local Council (LC) chairperson. The team constituted for TB screening at landing sites included: health workers, the landing site leadership (LC chairperson, VHTs, and any other leaders), and project staff. The team agreed on the most appropriate time and day of the week when the community members were likely to be available for the activity. Clear assignment of roles to the different members of the team proved important for efficient mobilization of the community and screening. The VHT, together with the community leadership, decided on the most appropriate venue. House-to-house mobilization was feasible since houses are very close to each other at landing sites. The health providers and laboratory personnel conducted the screening and collection and packing of sputum, respectively. On the scheduled date, the project team ensured that logistics (sputum containers, polythene bags for packaging samples, cotton wool, and a cold box with ice packs, gloves, and face masks) for TB screening were delivered at the screening venue. We applied the same algorithm for screening in congregate settings in the fishing communities.

#### Improved adherence to guidelines

According to the NTLP algorithm for diagnosis and management of TB for presumptives where MTB is not detected and for smear-negative suspects, further clinical evaluation and/or X-ray is required. We set out to increase the number of patients eligible for X-ray who got the X-ray**.** To increase TB case detection and eventual improvement in TB case notification, health workers from 10 health facilities were selected and trained in clinical diagnosis and use of X-ray to diagnose TB. The training lasted 5 days and was followed by two rounds of coaching/ mentorship by specialists in lung disease and radiology from the Makerere Institute of Lung Health. Coaching involved meeting with teams to review X-ray films and interpretation notes made by the health workers at each health facility. The specialist would make observations and compare their readings with the local health worker’s findings. At each facility, health workers were also supported to properly investigate patients clinically so they make the right clinical diagnosis.

## Methods

### Study design

This implementation study aimed to evaluate the effects of the QI-guided active case-finding intervention using a mixed methods approach to collect data on the different components of the intervention. The study assessed the intervention districts’ performance in identifying and screening vulnerable populations and diagnosing TB as well as documented health care provider experiences with the facility-led active case-finding intervention.

### Study period

The implementation of the intervention was initiated in November 2016 with pre intervention activities. Actual intervention started in December and continued until July 2017 in collaboration with the Ministry of Health NTLP and health facilities. During the initial phase of the study, baseline data was obtained on case notification to benchmark the intervention along with provider perceptions of QI-ACF.

### Study setting and population

The study involved collecting data about TB screening and diagnosis among the targeted populations namely inmates, fisher folks, HIV-positive clients, and TB case contacts. Sixteen health care providers (TB focal persons) from selected intervention facilities in eight districts were interviewed to obtain their perspectives on the implementation and feasibility of the QI-ACF intervention using a key informant interview guide. The same TB focal persons in the study sites were interviewed both at baseline and follow up for the purpose of consistent measurement. The 16 facilities were purposefully selected to represent the different levels of the health system.

### Data collection

For the targeted TB index patients, people living with HIV, incarcerated populations, and fishing communities, we collected data on the number screened, suspected, produced sputum, sputum evaluation, and positive cases from the health facility TB registers and case notification from the DHIS2. ACF data was collected from all 48 intervention health facilities. Case notification for intervention districts was analyzed for 6 months prior to the intervention, for the 6 months of the intervention (from December 2016 through May 2017), and post-intervention to determine if there were changes in notified cases of TB. To ensure all TB cases detected were notified, facility teams were guided to improve accuracy of TB records by synchronizing data between the TB unit and the laboratory registers. Tallies of the cases were made for those found in the laboratory register but are not in the TB unit register--un-notified cases. Such cases were then recorded in the unit TB register and then actively followed up so they were started on treatment. Provider experiences about the intervention were captured from 16 TB focal persons using a key informant guide covering major areas of the intervention, namely institutionalizing TB management at the facility, ACF, community awareness of TB, and challenges with implementing ACF for TB. The same respondents were interviewed at baseline and follow-up.

### Data analysis

Firstly, we analyzed TB case notification trends by using the routine TB surveillance data sourced from the district health information system for the intervention areas. We extracted trends in bacteriologically-confirmed cases, clinically diagnosed TB cases, and extra-pulmonary TB cases. We analyzed data points of 3-month intervals before and during the intervention. An additional 2 months of data (June–July 2017) was used to estimate the trend of cases after the intervention. The period-adjusted analysis enabled us to assess the effect of the intervention in terms of time. QI-ACF was an intervention event across different target groups. Thus we analyzed separately for each target group by number of suspects screened, suspected, produced sputum, sputum evaluated, and number of positives. Because of the nature of the data, simple statistics were used to describe the data in graphical form. Provider experiences about QI-ACF were documented and categorized into four themes, namely: institutionalizing TB management, TB case detection in QI-ACF compared to passive case-finding, community awareness of TB, and common challenges during QI-ACF. Thematic analysis supported by proportions, where applicable, was used to reveal provider experiences about QI-ACF.

### Ethical considerations

The intervention was reviewed for ethical considerations and approved by Mildmay-Uganda, Research Ethics Committee (MUREC) a local Institutional Review Board (IRB) and Uganda National Council of Science and Technology (UNCST). Administrative approval was obtained first from the Ministry of Health, District Health Officers and Facility In charge in the study districts. All QI-ACF activities were informed and voluntary.

## Results

### Change in TB case notification

**Fig. 2:**
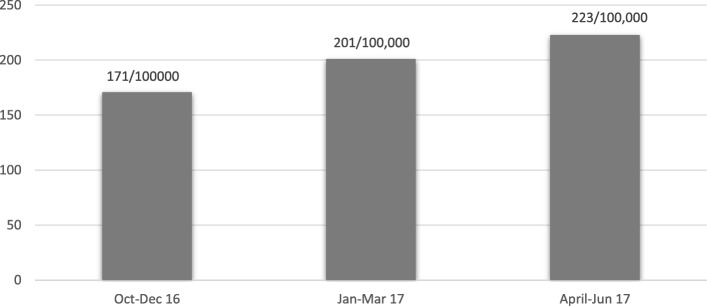
TB case notification in 10 intervention districts of Northern Uganda as extracted from DHIS2

Overall, TB case notification increased from 171 to 223 per 100,000 population between December 2016 and June 2017 (Fig. 2) in the 10 intervention districts. Analysis of DHIS2 data for Northern Uganda for 10 districts over the intervention period suggests a positive trend towards achieving the national target of 253/100000 population TB case notification. Results in Fig. 2 are based on new cases only and excludes relapses.

Overall in the 10 intervention districts of Northern Uganda during the intervention period of December 2016–May 2017, 214 cases were diagnosed through systematic TB screening during the intervention period, 59 of whom were diagnosed by X-ray and 41 of whom were diagnosed through improving the quality of data by ensuring all TB data tools are harmonized. The majority of TB-positive cases (114) were identified during active case-finding among the vulnerable populations: 41 TB cases were identified from among index TB patient contacts (6.1% positivity). Fishing communities yielded 27 TB cases and had the highest TB positivity rate at 6.8%. Prisoners accounted for the lowest TB-positivity rate at 2.3% (34 cases). Only 12 TB cases were diagnosed among HIV-positive clients at 21 selected health facilities, a 3.4% positivity rate.

#### TB positivity rate among sub groups of risky populations

**Fig. 3:**
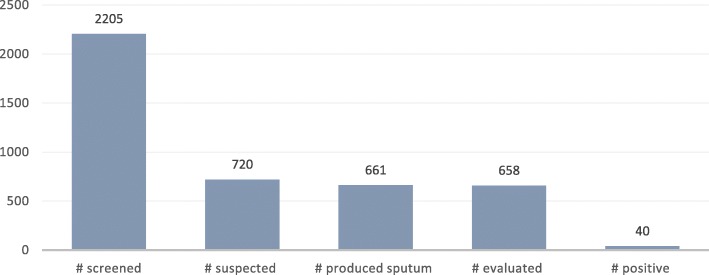
Number of TB cases among index case contacts

Out of the 2205 index TB case contacts screened, 720 (32.7%) were suspected. A high proportion of contacts who had been suspected produced sputum (661, 91.8%) and almost all evaluated (99.5%, 658). Samples collected from the community were delivered to the laboratory hubs for evaluation. In circumstances were the samples were many, they were uniformly distributed to other GeneXpert testing sites so that results could be ready in a short period of time. Forty TB-positive cases were diagnosed. Overall, a TB positivity rate of 6.1% was recorded among index TB case contacts through QI-ACF (Fig. 3).

**Fig. 4:**
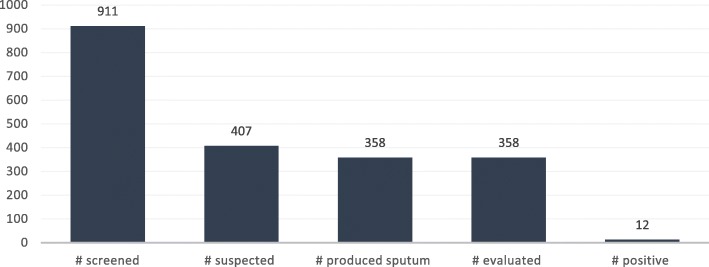
Number of TB cases among HIV clients in 21 facilities in Northern Uganda

HIV clients due for viral load were guided to produce sputum and sent for GeneXpert testing while the rest of the HIV patients were screened using the intensified case-finding tool. Out of 911 HIV clients screened in 21 facilities, 407 were suspected (44.7%). A significant number of those suspected produced sputum with the aid of health worker (358, 88%), and all of them were evaluated (100%), resulting in a TB positivity rate of 3.35%. The outcomes are contained in Fig. 4.

**Fig. 5:**
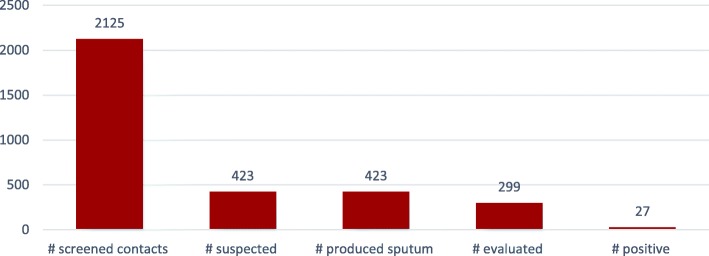
Number of TB cases among fishing communities

We screened 2125 persons in the fishing communities. This was enabled by the congestion in the fishing communities. Out of 2125 screened, 423 (20%) were suspected, and all of them produced sputum with the guidance of a health worker. Ninety-four percent (399) of fisher folks who produced sputum were evaluated, resulting in a TB positivity rate of 6.8% (27 cases). Results are contained in Fig. 5.

**Fig. 6:**
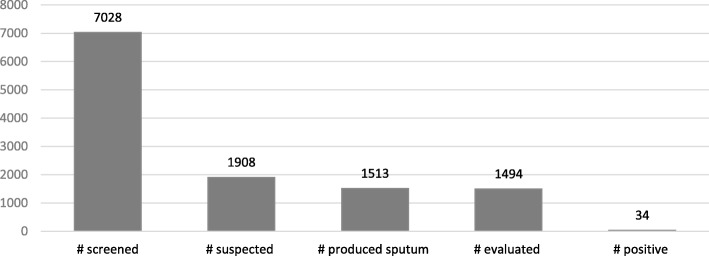
Number of TB cases among prisoners in 16 prisons in Northern Uganda

A total of 7028 prisoners were screened from 16 central and farm prisons in all of Northern Uganda. Out of those screened, 27% were suspected (1908) and 79.3% (1513) produced sputum. Sputum evaluation was done for the 1494 who produced sputum (98.7%), and 34 cases of TB were detected, resulting in a TB positivity rate of 2.3%. The results are contained in Fig. 6.

### Identification of HIV-positives among newly diagnosed TB-positives

MOH policy is to test all newly diagnosed TB patients for HIV (unless HIV positive status is already known). Of the 214new TB patients identified through the QI-ACF intervention during the period December 2016–May 2017, 20 (19.8%) were newly diagnosed with HIV, representing the HIV positivity yield among newly diagnosed TB patients from vulnerable populations.

### TB clinical and X-ray diagnosis results

The number of TB cases identified through clinical diagnosis and use of X-ray to diagnose TB increased from 34 out of 149 total TB cases in the quarter January to March 2017 to 94 out of 218 TB cases in April and May 2017, representing a 50% increase.

### Health care provider perspectives of QI-ACF

#### Institutionalizing TB management

Facility TB focal persons, when asked to describe the QI-ACF intervention, reported that it extended beyond the TB focal person, a move towards institutionalizing TB. They noted that health facility in-charges, health assistants, laboratory personnel, community linkage facilitators, and VHTs were involved in contact tracing, with each one playing different roles. For example, the VHTs screened and mobilized suspects ahead of the QI-ACF teams. Laboratory personnel collected and packed samples, while health care providers provided health education and screening of suspects. This represented a change from traditional TB programming, where all TB work was left to specialized TB health workers.

#### QI-ACF resulted in more TB case detection than passive case-finding

All providers (100%) indicated that TB diagnoses from QI-ACF activities were greater than passive case-finding and occurred over short intervals of time. In line with this advantage, providers associated QI-ACF with identifying increased number of presumptive cases and therefore enabling screening of larger number of suspects that would result into high TB positivity rate.

#### Community awareness of TB

We sought information on the provider perspectives about community awareness for TB through key informant interviews with TB focal persons. Our findings showed that 81% of the providers perceived low awareness of TB in the community prior to the intervention. However, after the intervention, most providers (96%) cited QI-ACF as one of the sources of information about TB in the community. Other sources of information included the integrated community outreaches for HIV Counseling and Testing, immunization, cervical cancers screening, deworming and antenatal care (10%), radio messages on TB (2%) and churches (2%).

#### Common challenges during QI-ACF

Our study documented common challenges in implementing QI-ACF from the provider key informant interviews. Some related to the logistics of moving TB samples and making lab supplies available where needed: an insufficient number of hub riders slowed down the movement of samples; there were deficiencies in the supply of reagents, leading to stock- outs at various testing points; and there were times when samples were rejected predominantly on account of being salivary because of poor inducement, day-time sample, and low community awareness about TB. Due to multiple challenges, the X-ray machines were only functional for half of the intervention period in some facilities. Despite this challenge a 50% increase in patients diagnosed through clinical diagnosis and use of X-ray was observed.

## Discussion

The QI-ACF intervention had quality improvement principles and tools embedded in its implementation and operated at all three levels of the health system in Northern Uganda: above facility, facility, and community levels. Facility-level activities involved synchronizing TB data between clinical and lab registers, two-tier mapping of contacts, ICF, checking quality of sputum samples, completing health management information system (HMIS) tools, sample evaluation, giving results to patients, starting TB patients on treatment, leading and coordinating screening in vulnerable populations, and implementing clinical and/or X-ray evaluation of suspected cases who were smear-negative. Community-level activities involved: tracing presumptive patients lost to follow-up, tracing targeted contacts of TB patients, sputum sample collection, community mobilization within the targeted vulnerable population, and screening and health education within the vulnerable groups. Above-facility activities included: selecting intervention facilities and engaging regional, district, and facility leaders.

Measuring direct yield would be the first step to assess the outcome of this QI-ACF intervention [20]. Monthly adjusted notification data from DHIS2 and data on GeneXpert utilization in Northern Uganda [12] indicated that case notifications substantially increased during the intervention period as compared to historical baseline cases in intervention and non-intervention sites. The positive improvements in case notification in intervention sites over the intervention period attributed to QI-ACF imply that replicating the same model could lead to increased numbers of TB cases diagnosed.

Our results were consistent with several studies and reports that documented improved case notification through ACF. For instance, in Ethiopia, an intervention package that utilized community based ACF initiatives doubled case notification of smear-positive TB cases compared to the pre-intervention period and to the control areas [21]. Similar findings were found in India, where a project using community-based ACF and awareness strategies led to 11% increase in smear-positive notifications compared to pre-intervention period [22]. Such increases might be attributable to various factors, including a large number of subjects screened during ACF exercises relative to routine passive case-finding as well as the tailored intervention approach designed to reach the specific vulnerable populations in question.

The yield of screening increases when the search is focused in high-risk populations [18]. QI-ACF targeted high-risk groups for pulmonary TB, such as prisoners, people with HIV, fisher folks, and contacts of index patients. The outcome implies that the number of cases for TB could be increased if high-risk groups for TB are targeted deliberately through outreaches.

QI-ACF in the intervention sites involved different human resource cadres from the health facility and the community beyond the TB focal person. Health facility in-charges, health assistants, laboratory personnel, community linkage facilitators, and VHTs were common participants in contact tracing. This engagement of a broader group of health personnel suggests that the intervention produced a shift in provider perception about TB from being centered on one focal person, to a concern of the wider health facility team. This is a step towards building health systems and institutionalizing TB in manner that is participatory as well as raising the sensitivity of all providers to their role in detecting TB.

The results show an increase in clinical diagnosis of TB over the intervention period. Our baseline TB CNR in the intervention districts is 171 higher than the national estimated case notification rate of 136 per 100,000. A further increase in the TB CNR in the intervention districts suggests the effect of the ACF model on case notification due the observed change during the intervention period only.

Although the algorithm clearly requires X-ray for negative presumptive cases, this was rarely done in Northern Uganda. However, increased notification of TB cases was observed following provider training in clinical diagnosis in the intervention sites. Programmatically and technically, subsequent increases in clinically diagnosed TB following the intervention would result in additional cases notified over time.

QI-ACF among vulnerable populations also implies a possible reduction in new infections. Although this could not be ascertained in the short time period of this intervention study, we posit that the notification and treatment of cases would reduce transmission and incidence that would otherwise continue without any intervention. This calls for further studies to determine prevalence and transmission profiles in the study sites and communities in the catchment areas of the sites.

The direct involvement of laboratory staff in QI-ACF enabled the processing of samples to be fairly speedy and allowed results at slightly shorter intervals, thus closing the gap between screening and initiation of treatment.

There was mixed opinion about awareness of TB at baseline. We observed that the QI-ACF intervention played a multi-functional role of health education and screening suspects while raising community sensitivity to TB. According to the Ministry of Health [6], ignorance about TB is a barrier to care-seeking, even among those with possible manifest symptoms. QI-ACF initiatives should therefore integrate health education as a key activity while entry points should be used as a means of reaching out to people with messages on TB in absence of awareness programs for TB.

## Conclusions

Targeting should be applied in TB interventions to improve yield and use resources more efficiently. We recommend targeting, for example using the pareto rule to guide targeting districts and facilities with 80% of the missing cases, targeting populations to focus on the vulnerable, and targeting communities where 80% of the missing contacts are. Quality improvement should continue to be applied to come up with new innovations to identify undiagnosed TB patients. Non-traditional vulnerable populations like fishing communities were identified through QI team meetings. We recommend functionalizing QI teams to address the case notification gap at each health facility. A product of the iterative testing cycles of the QI methodology was to repeat screening for TB in the urban but not in the rural prisons. This was because the teams found more presumptive TB cases and positive cases in urban than rural prisons. We recommend further research to document and explain this observation.

A screening tool should be provided for vulnerable populations to guide health workers to identify presumptive cases. The screening tool we used directs all HIV patients eligible for viral load to provide sputum for evaluation. This was tested as a quality improvement change to simplify the management of people with HIV, especially with increasing workload at the health facilities following the roll-out of the test and treat guidelines for people with HIV and differentiated service delivery models which decrease the number of times the HIV patient is in contact with the health worker. We recommend implementation of this contextually appropriate concept, while fine tuning it to improve its yield. We found a significant number of patients using X-ray in spite of the multiple challenges associated with functionalizing the X-ray machine. Use of X-ray is recommended and should be accompanied by a quality assurance program to minimize misdiagnosed smear- negative pulmonary TB. Data improvement for TB, specifically synchronizing laboratory and TB registers, is another critical and timely recommendation, especially in the current context of recently adding TB to the HMIS in Uganda.

## Additional files

Additional file 1: Algorithm for TB screening in close contacts to TB patients (DOCX 67 kb)

Additional file 2: Algorithm for TB screening in congregate settings (DOCX 94 kb)

Additional file 3: Algorithm for TB screening in the HIV-positive population (DOCX 58 kb)

## Abbreviations

ACF: Active Case-Finding
ASSIST: USAID Applying Science to Strengthen and Improve Systems
DHIS: District health information system
DOTS: Directly observed therapy short course
HC: Health center
HIV: Human immunodeficiency virus
HSSP: Health sector strategic plan
ICF: Intensified case-finding
IRB: Institutional review board
NTLP: National TB and Leprosy Control Program
PEPFAR: President’s Emergency Plan for AIDS Relief
QI: Quality improvement
S&S: Signs and symptoms
SDG: Sustainable development goal
TB: Tuberculosis
UNCST: Uganda National Council of Science and Technology
US: Ultra sound
USAID: United States Agency for International Development (USAID), Uganda
VHT: Village Health Teams
VL: Viral load
