# Supplementary material for: Improving TB case notification in northern Uganda: evidence of a quality improvement-guided active case finding intervention
Source: BMC Health Serv Res. 2018 Dec 12;18:954. doi: 10.1186/s12913-018-3786-2 (PMC6292080; doi:10.1186/s12913-018-3786-2)
Supplement: Supplementary file 1 — Algorithm for TB screening in close contacts to TB patients (DOCX 67 kb) [file 12913_2018_3786_MOESM1_ESM.docx]

**CLOSE CONTACTS TO TB PATIENTS**


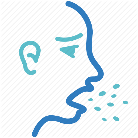


**Assess with ICF job aid/Form**

**Cough for 2 weeks**

**No cough and no to other TB S&S**

**No cough; but has other S&S of TB**


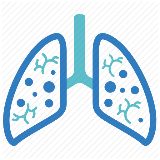


**TB Suspect**

**Refer to health facility for clinician’s further evaluation**

**Rescreen for TB when next visits a health facility**

**Presumptive TB case, collect sputum and follow MoH TB diagnostic algorithm**
