# Supplementary material for: Improving TB case notification in northern Uganda: evidence of a quality improvement-guided active case finding intervention
Source: BMC Health Serv Res. 2018 Dec 12;18:954. doi: 10.1186/s12913-018-3786-2 (PMC6292080; doi:10.1186/s12913-018-3786-2)
Supplement: Supplementary file 2 — Algorithm for TB screening in congregate settings (DOCX 94 kb) [file 12913_2018_3786_MOESM2_ESM.docx]

**PERSONS IN CONGREGATE SETTINGS**

**Assess with ICF job aid/Form**


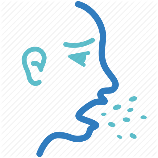


**Cough**

**No cough; but has other TB S&S**

**No cough and No to other TB S&S**

**Refer to clinician to assess for extra pulmonary TB Disease**

**Screen for TB after 3 months**

**TB Suspect**


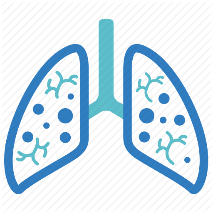


**TB disease excluded**

**Extra pulmonary Disease not excluded**

**Investigate to exclude TB; X-ray, US, lung biopsy**

X-ray

**Presumptive TB Case. Follow MOH TB Diagnostic algorithm**

NB: Persons in congregate settings should be encouraged to have HIV testing at TB screening
