# Supplementary material for: Improving TB case notification in northern Uganda: evidence of a quality improvement-guided active case finding intervention
Source: BMC Health Serv Res. 2018 Dec 12;18:954. doi: 10.1186/s12913-018-3786-2 (PMC6292080; doi:10.1186/s12913-018-3786-2)
Supplement: Supplementary file 3 — Algorithm for TB screening in the HIV-positive population (DOCX 58 kb) [file 12913_2018_3786_MOESM3_ESM.docx]

**Due for VL testing?**

**Assess with ICF job aid/Form**

**HIV POSITIVE PERSONS**

**NO**

**Collect sputum for GeneXpert testing**

**YES**

**Any**

**Cough**

**No cough; but has other TB S&S**

**No cough and No other TB S&S**

**Investigate as necessary X-ray, US, lung biopsy**

**Clinician for further assessment for TB**

**Screen for TB at next Clinic visit**


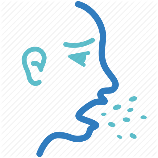


**Collect Sputum**

**Presumptive TB Case. Follow MOH TB Diagnostic algorithm**
